# Sequential Codoping Making Nonconjugated Organic Radicals Conduct Ionically Electronically

**DOI:** 10.1002/smsc.202100081

**Published:** 2021-10-04

**Authors:** Yerin Jo, Ilhwan Yu, Jaehyoung Ko, Ji Eon Kwon, Yongho Joo

**Affiliations:** ^1^ Institute of Advanced Composite Materials Korea Institute of Science and Technology (KIST) 92 Chudong-ro Bongdong-eup Wanju-gun Jeonbuk 55324 Republic of Korea; ^2^ Department of Nanoconvergence Engineering Jeonbuk National University 567 Baekje-daero, Deokjin-gu, Jeonju-si Jeonbuk 54896 Republic of Korea; ^3^ Department of Chemistry Hanyang University 222 Wangsimni-ro Seoul 04763 Republic of Korea; ^4^ Department of Chemical and Biomolecular Engineering and KAIST Institute for Nano Century Korea Advanced Institute of Science and Technology (KAIST) Daejeon 34141 Korea

**Keywords:** codoping, mixed ionic–electronic conductor, organic radical molecules

## Abstract

Mixed conduction through both ionic and electronic pathways in an organic radical has received enormous attention recently, owing to its high conductivity and exceptional processibility amenable to future organic electronics. While the majority of previous works have centered on polymeric systems, the study on the mixed conduction in a small molecular radical has gained less attention despite its enormous potential. Herein, a study on the mixed conduction behavior of such system, 4‐substituted 2,2,6,6‐tetramethylpiperidyl‐1‐oxy (4‐hydroxy TEMPO, HT), via sequential codoping with an ionic dopant, lithium bis(trifluoromethanesulfonyl)imide salt (LiTFSI, LT), and an electronic dopant, 2,3,5,6‐tetrafluoro‐7,7,8,8‐tetracyanoquinodimethane (F4TCNQ, FT), is presented. It is found that the coupling between the components plays an important role in determining the total conductivity, in which a maximum conductivity of ≈10^−4^ S cm^−1^ was obtained for a HT/LT/FT mixture. A systematic study to connect the physical changes associated with doping and the observed mixed conductivity is provided. It is believed that these findings establish a starting point to study mixed conduction behaviors in small molecular organic radical systems in general, ultimately targeting next‐generation organic electronic devices and batteries.

## Graphical abstract

Herein, mixed conduction through both ionic and electronic pathways in a small molecular organic radical is studied. Molecular doping strategy for enhanced mixed conductivity is presented, where a maximum conductivity of ≈10^−4^ S cm^−1^ is obtained for a ternary mixture. A systematic study to connect the physical changes associated with doping and the observed mixed conductivity is also provided.

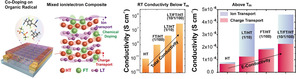

## 1 Introduction

Transporting charges through both electronic and ionic pathways in an organic compound has been emerging as a powerful strategy of diversifying and enhancing the functionality of conventional devices, and directed significant attention to various device applications such as organic electrochemical transistors for circuits and neuromorphic computing,^[^ ^1^ ^]^ chemical sensors,^[^ ^2^ ^]^ energy storage,^[^ ^3^ ^]^ light‐emitting electrochromic devices,^[^ ^4^ ^]^ and ion pumps.^[^ ^5^ ^]^ Key transport mechanisms under which a given device attains target‐specific functionalities have been discussed, where the ionic–electronic coupling has indicated a significant role in determining the overall transport properties as the two individual pathways.^[^ ^6^ ^]^ Other than these fundamental transport processes, the so‐called structure–property relationship that involves the practical aspects of device fabrication, has also shown a great influence on overall device performances. Our current understanding of the interrelationship between these fundamental processes and their processing, however, is only incipient, and any future effort seeking improvements on the device performances or its multifunctionalization is expected to contribute significantly to the general understanding of the field.

For organic systems that have been popular in these areas, either electronic and ionic doping has been the main strategy to improve the charge transport relative to the pristine component.^[^ ^6^, ^7^ ^]^ A well‐known example is poly(3,4‐ethylenedioxythiophene) polystyrene sulfonate (PEDOT:PSS), where PSS acts as a macromolecular ionomer for enhancing the ionic conduction of PEDOT.^[^ ^8^ ^]^ Often added to this system was an ionic dopant, bis(trifluoromethanesulfonyl)imide lithium salt (LiTFSI, LT), which tuned the macroscopic conductivity of PEDOT:PSS further by increasing p‐doping density.^[^ ^9^ ^]^ Another popular example of doping an organic system is poly(3‐hexylthiophene‐2,5‐diyl) (P3HT), where 2,3,5,6‐tetrafluoro‐7,7,8,8‐tetracyanoquinodimethane (F4TCNQ, FT) was utilized as a small molecular electronic dopant.^[^ ^10^ ^]^ Similar to the case of PEDOT:PSS, further addition of the ionic dopant LT to this system further led to an increase in the ionic conductivity.^[^ ^11^ ^]^ Switching between the types of dopants and hence the two conduction pathways in these multicomponent systems has enabled the fundamental charge transport processes of these systems and their interrelationship easier to investigate. The majority of the previous works in these areas, however, have focused on conjugated systems as a base material either in the form of macro and small molecules.

Organic radicals are a group of conducting (macro‐)molecules that feature open‐shell chemistry with a nonconjugated molecular structure in its entirety, where the charge transfer occurs through a reversible redox hopping between the active radical sites.^[^ ^12^ ^]^ Because of their physical characteristics that are fundamentally different from the conventional organic conductors, they feature exceptional processibility, good synthetic yields, and optical transparency while possessing relatively high electrical conductivity.^[^ ^13^ ^]^ In the previous work, we reported the charge transport characteristics of a model macromolecular compound of such, poly(4‐glycidyloxy‐2,2,6,6‐tetramethylpiperidine‐1‐oxyl) (PTEO), where we pioneered the concept of radical conduction and further developed it via ionic doping utilizing LT.^[^ ^13^, ^14^ ^]^ In a consecutive report, we expanded the idea of the radical conduction to a small molecular radical, 4‐hydroxy‐2,2,6,6‐tetramethylpiperidin‐1‐oxyl (hTEMPO, HT),^[^ ^15^ ^]^ and witnessed an interesting and unique conduction behavior that is phase‐dependent due to its specific molecular arrangements upon phase transition.^[^ ^15^ ^]^ However, further efforts on improving such small molecular radicals as a novel conducting system for future organic electronics, since then have been elusive.

In this contribution, we expand the conventional idea of molecular doping to this emerging class of conducting material, organic small molecular radicals, via electronic and ionic (co‐)doping where the mixed conduction behavior is effectively modulated on demand. Briefly, to a model radical HT, we first introduce an electronic dopant FT to facilitate its electronic charge transport, which is followed by separate ionic doping by LT to enhance the ionic conductivity. Ultimately, we explore codoping of HT by both dopants, where we report an interplay between the components owing to the effective coupling between the two conduction pathways. In addition, we discuss the interconnection between the physical characteristics of the doped systems and the observed mixed conductivity. We believe our findings represent a substantial shift from the conventional understanding of molecular doping, establishing a paradigm of doping organic radical conductors necessary for the development of next‐generation organic electronics.

## 2 Results and Discussions

**Figure 1:**
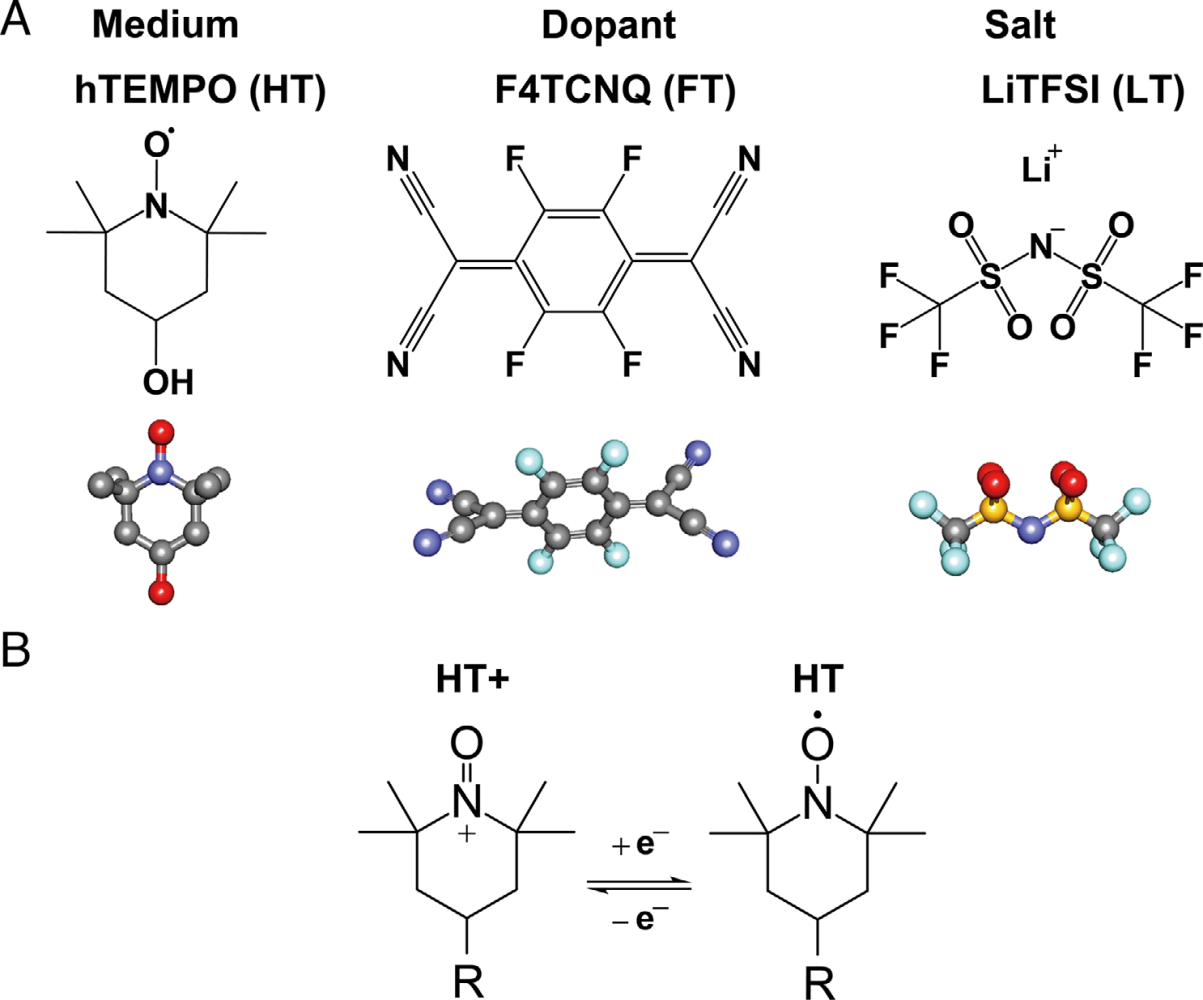
A) Chemical structure of charge transporting medium hTEMPO (HT), an electrical dopant F4TCNQ (FT), and an ionic dopant LiTFSI (LT) used in this study for the mixed conduction. B) Reduction–oxidation self‐exchange reaction that occurs from the nitroxides of HT, for radical conduction.

The organic small molecular radical utilized in this effort, HT, consists of a stable nitroxide radical linked to a hydroxyl functional group. It transfers charge as the nitroxide oxidizes to give an oxoammonium cation, through the electronic self‐exchange reaction that occurs between the active species in close proximity.^[^ ^15^ ^]^ Unlike its polymeric analog, PTEO, where the charge transfer occurs between the pendant groups that are adjacent, HT provides a relatively higher density of the redox‐active sites owing to the absence of a backbone structure that is often electrically insulating and potentially represents the full potential of the open‐shell chemistry.^[^ ^15^ ^]^ For both electronic and ionic doping of HT, we employ the conventional dopants frequently used in organic electronics and batteries as model compounds. Specifically, we adopt FT for the electronic doping of HT, which is a very effective p‐type dopant owing to its favorable lowest unoccupied molecular orbital (LUMO) energy level.^[^ ^16^ ^]^ For the ionic doping of HT, we introduce LT that has been popular as a highly stable Li‐ion source in electrolytes for batteries.^[^ ^17^ ^]^ The molecular structures of HT, FT, and LT, and the radical conduction mechanism in HT are summarized in **Figure** **1**A,B. The incorporation of these molecular dopants into HT results in three different types of mixtures, each with varying compositions. These are the mixtures of HT and FT (hereafter will be referred to as FT/HT), HT, and LT (LT/HT), and the mixture of HT with both dopants (LT/FT/HT). Whenever necessary, the molar ratio of the components in the mixture will be specified in parentheses (e.g., a 1:1 molar mixture of HT and FT is denoted as FT/HT(1/1)).

**Figure 2:**
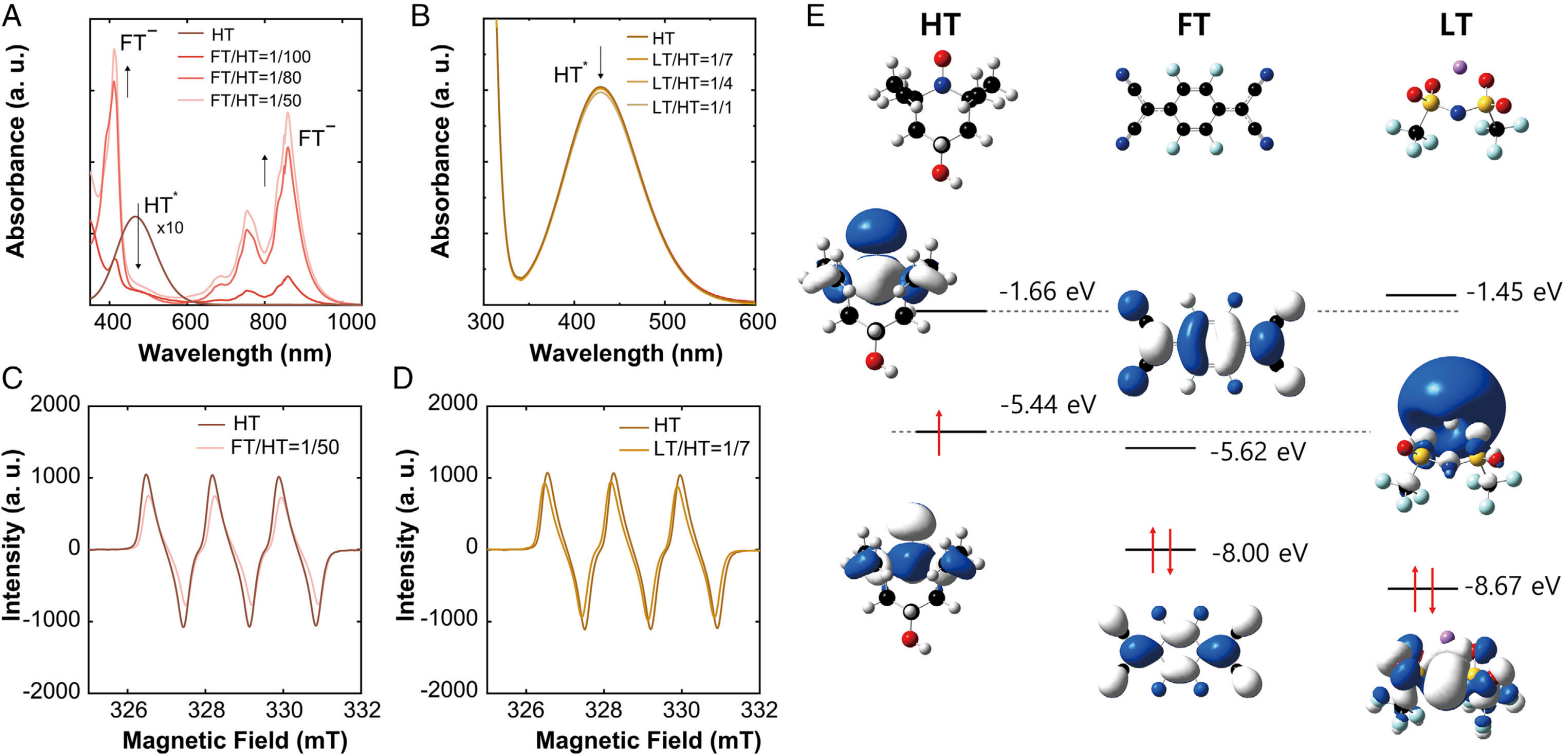
Spectroscopic analysis of the doping effect on HT. A,B) Absorption spectra of HT doped with different molar ratios of dopants, (A) FT (FT/HT of 1/100, 1/80, and 1/50) and (B) LT (LT/HT of 0, 1/7, 1/4, and 1/1). C,D) Electron paramagnetic resonance spectra of HT, and the spectra of HT doped with a selected molar ratio of dopants, (C) FT/HT(1/50) and (D) LT/HT(1/7). E) Unperturbed energy diagram illustrating the electronic states of HT, FT, and LT. Electron transfer from HT to FT is preferred by the sizable energy offset between the ionization energy (IE) of HT (−5 eV) and the electron affinity (EA) of FT (−5.2 eV).

We first explore the effect of each dopant on the electronic conduction of HT via a set of spectroscopic analyses, and the results are summarized in **Figure** **2**. Figure 2A shows the UV–vis spectra of a series of FT/HT. Originally, pristine HT features its strong absorption at ≈500 nm due to the presence of nitroxide (NO˙) (see Figure 2B).^[^ ^18^ ^]^ However, the spectra after the electronic doping (i.e., adding FT) were dominated by a new group of peaks at ≈411, 682, 753, and 858 nm, due to the presence of the anionic (radical) species of FT, namely, FT˙^−^ and FT^2−^.^[^ ^19^ ^]^ Here, the spectrum of the pristine FT was omitted for clarity, as it has no identifiable absorption in this spectral region. The maximum absorbance observed for FT/HT increased monotonically with an increasing molar ratio of FT, from FT/HT(1/100) to FT/HT(1/50), showing a plateau afterward. Figure 2B shows the UV–vis result of HT after the ionic doping (i.e., adding LT) at different molar ratios. The peak at ≈500 nm from NO˙, showed a small decrease in the peak intensity compared with that of the pristine HT (Figure 2B). Figure 2C,D compare the electron paramagnetic resonance (EPR) spectra of FT/HT and LT/HT at different molar ratios, respectively. With the dramatic changes observed in the UV–vis spectra of FT/HT (Figure 2A), the addition of only a small amount of FT to the pristine HT (e.g., FT/HT(1/50)) resulted in an appreciable decrease in the peak intensity in the EPR spectrum, with a small peak shift (Figure 2C). A similar decrease in the peak intensity was also achieved by the addition of relatively large amounts of LT to the pristine HT (LT/HT(1/7)) (Figure 2D). From the generation of the (di‐)anionic species of FT in the UV–vis results, it can be deduced that the addition of FT to HT led to the formation of the cationic species, HT^+^, by means of electron transfer. The resultant HT^+^ can then facilitate the overall electronic conduction of HT, by providing active sites for the self‐exchange redox reaction. The formation of HT^+^ is supported by the decrease in the EPR peak intensity of FT/HT(1/50) (Figure 2C). Further, we conducted a density functional theory (DFT) calculation on the energy levels of each, where it showed a facile electron transfer from a singly occupied molecular orbital of HT (SOMO, −5.44 eV) to the LUMO of FT (−5.62 eV) highly probable (Figure 2E) **(**calculation details are provided in the Supporting Information). Noteworthy is that the ionic doping of HT also resulted in the generation of HT^+^, providing indirect evidence on the possible ionic–electronic coupling between the two species. This effect, commonly known as the ionic–electronic coupling in the literature, has also been found in the ionic doping of its polymeric analogue (i.e., PTEO) previously.^[^ ^14^ ^]^ Note, however, that the amount of such coupling was achieved only when the amount of the dopant was comparable to that of the pristine HT (LT/HT > 1/7).

**Figure 3:**
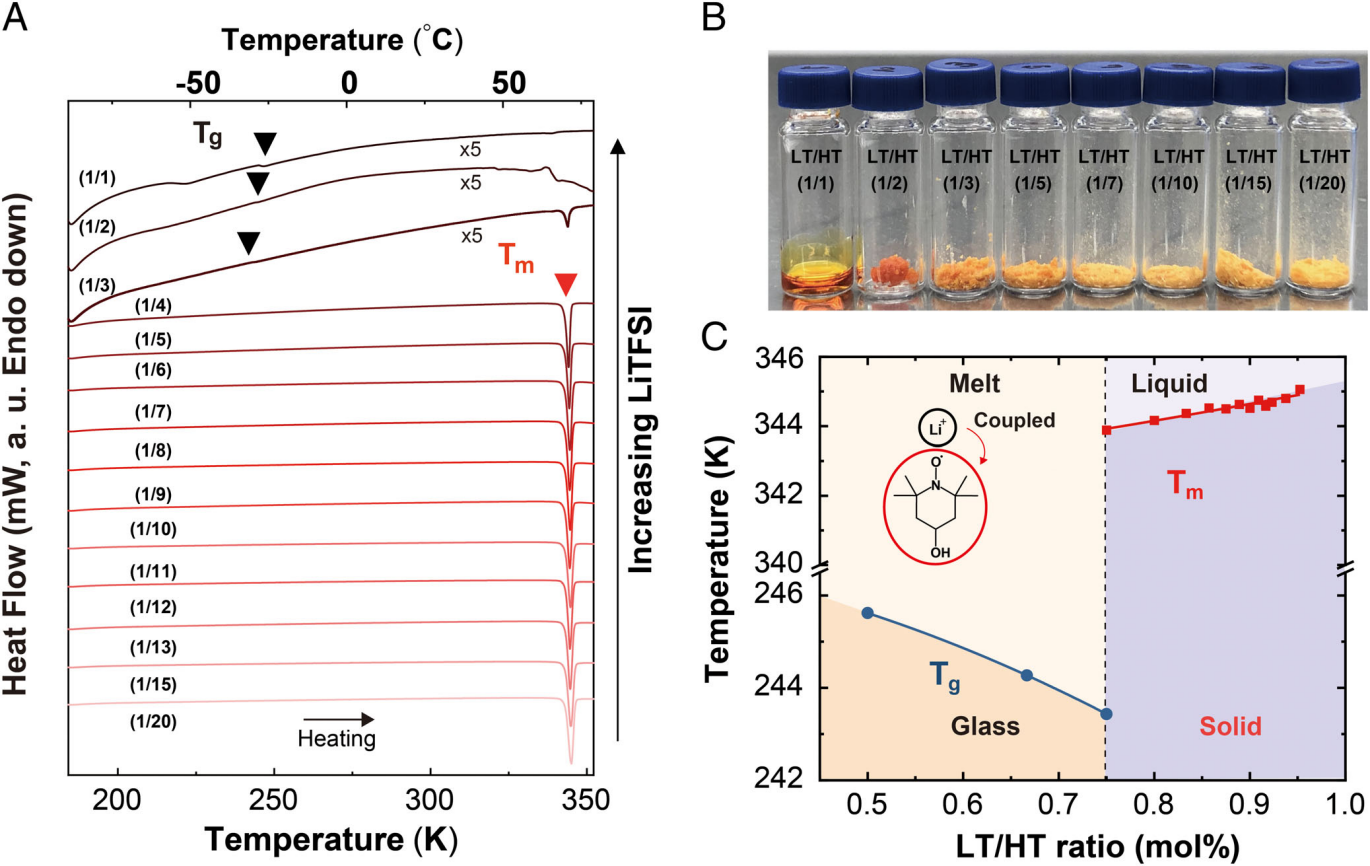
Physical changes were observed in LT/HT at different molar ratios. A) A series of DSC thermograms showing the change in the thermal properties of LT/HT, as compared with those of pristine HT. Molar ratio tested includes: LT/HT 1/1, 1/2, 1/3, 1/4, 1/5, 1/6, 1/7, 1/8, 1/9, 1/10, 1/11, 1/12, 1/15, and 1/20. Corresponding *T*_g_ and *T*_m_ are marked by the arrowheads. B) Photographs of a series of LT/HT used in the DSC study, displaying various physical states depending on the mixing ratio. C) Experimental phase diagram of LT/HT constructed from the DSC results.

For more direct evidence on the ionic interaction and the coupling between LT and HT, we then proceeded with a different set of physical characterization methods. In our previous work, we reported the charge transport of the model compound HT to be highly phase‐dependent due to the close proximity requirement of the redox hopping.^[^ ^15^ ^]^ Based on this, we hypothesize that the ionic doping and the corresponding ionic transport in our case should also be highly dependent on the phase of the mixture. **Figure** **3** summarizes the differential scanning calorimetry (DSC) results for LT/HT at different compositions. At a low LT/HT ratio (i.e., a small amount of LT addition), the mixture showed a thermogram similar to that of the pristine HT (Figure 3A). As the relative amount of LT increases, the observed melting temperature (*T*_m_) showed an appreciable decrease. Interestingly, the mixtures of high LT/HT ratio (i.e., a large amount of LT addition) featured a glass transition temperature (*T*_g_), which created a waxy solid phase or even a liquid phase of LT/HT (Figure 3B,C). The experimentally acquired phase diagram in Figure 3C summarizes the overall phase behavior of LT/HT at different compositions. Overall, the systematic decrease in the *T*_m_ is attributed to the melting point depression effect that commonly occurs in systems containing ionic species.^[^ ^20^ ^]^ Noteworthy is the formation of the waxy solid or the liquid phase for LT/HT mixtures having compositions above LT/HT(1/3), which is somewhat counterintuitive considering that each of them was in the form of solid when separated. This physical state of LT/HT has been described as “a solvated ionic liquid” or “a redox‐active supercooled liquid” in previous studies. The phase has been shown to enhance the ionic conduction of the system remarkably, owing to its liquid state that facilitates the movement of the ionic carriers in the medium.^[^ ^20^ ^]^

**Figure 4:**
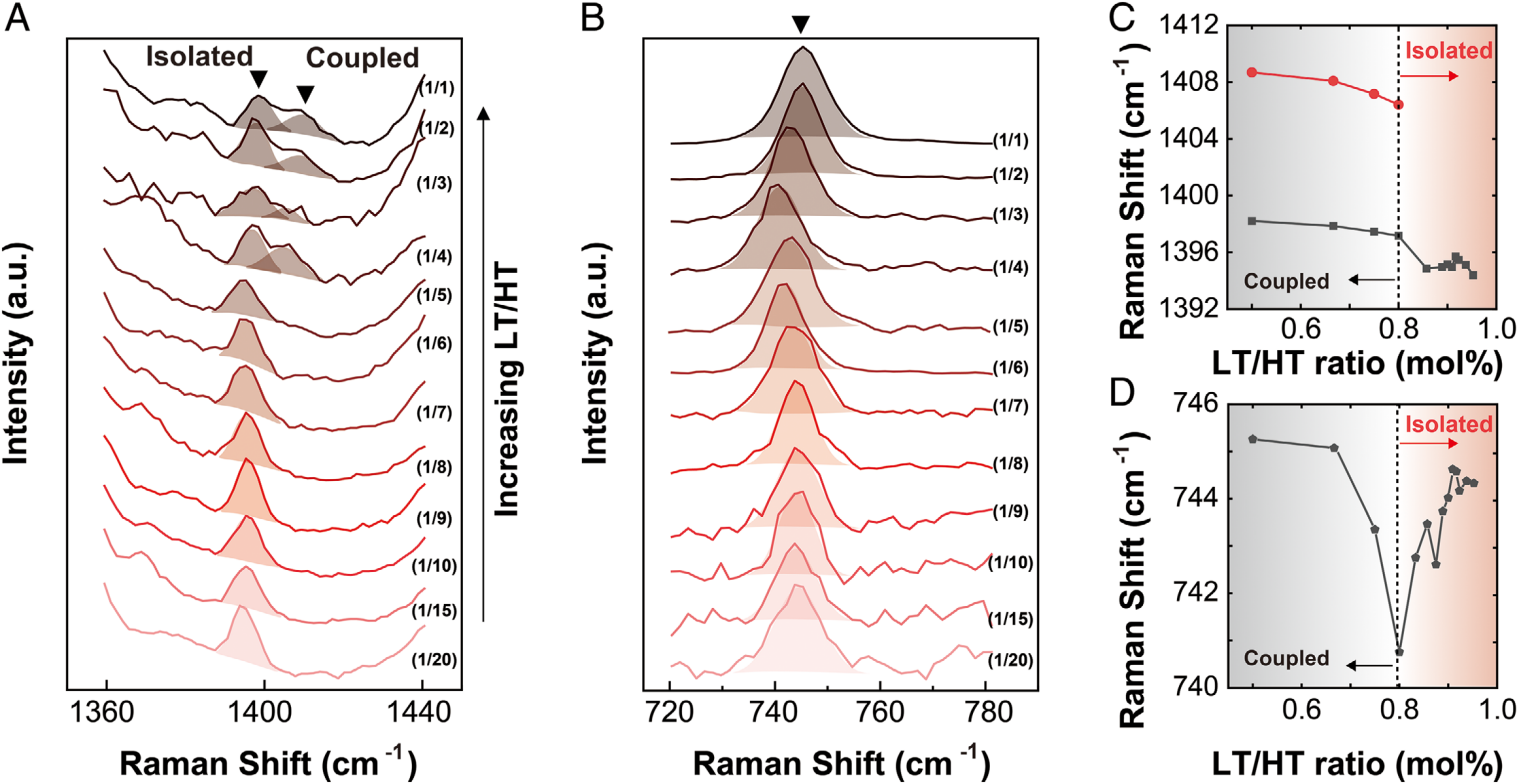
Raman spectra of a series of LT/HT at different molar ratios. A,B) Raman spectral regions of (A) NO. and (B) TFSI^−^ species. C,D) Corresponding shifts in the Raman shift observed in (C) NO., and (D) TFSI^−^ spectral regions.

Building upon constructing the experimental phase diagram of LT/HT at different compositions, we then sought direct evidence of the ionic interaction or coupling in these mixtures via Raman spectroscopy, and the results are summarized in **Figure** **4**. Figure 4A,C shows the spectral region of NO˙ stretching vibration of HT, where the pristine HT shows its absorption maximum at 1386 cm^−1^.^[^ ^20^ ^]^ Interestingly, as we increase the amount of LT in LT/HT, there appeared a second peak at 1405 cm^−1^ for all the mixtures having a molar ratio at or above 1/4. We observed a similar trend in the peak region of TFSI^–^ (≈740 cm^−1^), where it showed a point of transition at the molar ratio around 1/4 (Figure 4B,D). Specifically, starting from the peak of pristine TFSI^−^ at 744 cm^−1^, the peak initially showed a systematic red‐shift to 741 cm^−1^ as LT increased. It was followed by a systematic blue shift from 741 to 746 cm^−1^, at a molar ratio ≥1/4. Overall, we attribute the appearance of the peak at 1405 cm^−1^, and the later blue‐shift observed in TFSI^–^, to the formation of a new ion pair between HT and LT.^[^ ^20^ ^]^ Specifically, the peak at 1405 cm^−1^ can be assigned to be a coupled species, (Li‐HT)^+^, which coexists with the free radicals in the mixture.^[^ ^20^ ^]^ Regarding the peak shift observed, the initial red‐shift is likely due to the decreasing crystallinity as the mixing ratio increases, while the blue‐shift indicates the change in the nature of the ionic interaction, presumably due to the interaction of HT with the new ionic species (i.e., (Li‐HT)^+^).^[^ ^20^, ^21^ ^]^ We argue that this observation provides direct evidence on the ionic–electronic coupling that occurs between LT and HT in our system. Further, we connect the formation of the ion pair to the phase transition observed in the DSC results (Figure 3). This is based on the experimental observation that 1) the DSC results showed the phase transition at a molar ratio that nearly coincides with that from the Raman results, and that 2) the appearance of the coupling observed in Raman occurs within through a very narrow range of composition, which is highly likely to be associated with a sharp physical change like phase transition. Physically, the results above can be explained as the diffusion of the ionic species (either Li^+^ or (Li‐HT)^+^) facilitated by the formation of the waxy solid phase, as opposed to the glassy state at lower LT content that largely limits such effect. This is also in line with the previous idea that the increased molecular motion within a waxy solid material enhances the conduction of a radical system.^[^ ^15^ ^]^ In addition, it is expected that the strong coupling between HT and LT may form a local order in transferring charges, further enhancing the conduction through the ionic pathway.^[^ ^13^ ^]^ Overall, the observation in the DSC and Raman results strongly indicates a significant cross‐talk between HT and LT in our system, where LT responds to the phase behavior of HT in the form of ionic interaction or coupling. Of specific note is that the waxy solid or liquids observed in our study showed high stability over a wide temperature range in the air, and are expected to be useful as regular ionic conductors (vide infra).

**Figure 5:**
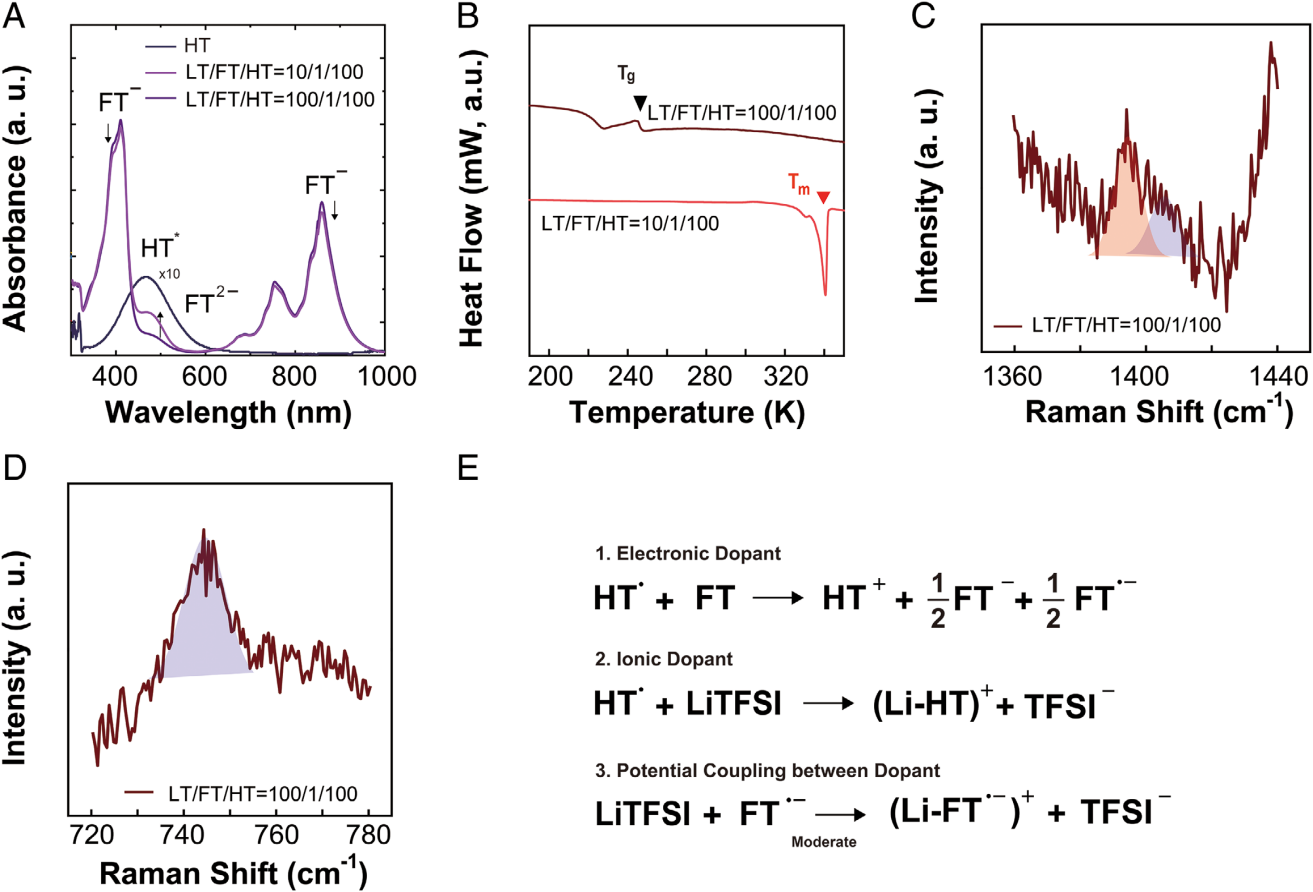
Characterization of the codoping system (LT/FT/HT). A) UV–vis spectra, B) DSC thermogram, and C,D) Raman spectra of the selected composition of LT/FT/HT. E) Possible interactions or coupling in LT/FT/HT system.

Having established the relationship between HT and each dopant, we then proceeded onto the codoping of HT by both FT and LT at selected molar ratios, to assess possible interaction or (de‐)coupling between each component that was not present in separate systems. The molar ratio of choice was LT/FT/HT(10/1/100) and LT/FT/HT(100/1/100), which considers the balance between the conduction through each transport pathway. **Figure** **5** summarizes the physical characteristics of the two ternary mixtures as observed by the aforementioned methods. UV–vis spectra of the ternary systems in Figure 5A, displayed spectral patterns dominated by the presence of the anionic (radical) species of FT, as in the case of FT/HT. Specifically, there appeared a similar group of peaks at ≈411, 682, 753, and 858 nm owing to the presence of FT˙^−^ and FT^2−^, with only a minor change in the peak intensity distribution. Similarly, DSC of LT/FT/HT(100/1/100) and LT/FT/HT(10/1/100) showed their characteristic *T*_g_ and *T*_m_ at −26.5 and 70 °C, respectively (Figure 5B). Compared with LT/HT(1/1) and LT/HT(1/10), the shifts in *T*_g_ and *T*_m_ ((−25.5) and (71.5 °C), respectively) of the ternary mixtures were relatively small and were within the experimental error. Figure 5C,D show the Raman spectra of the ternary mixtures. Here, LT/FT/HT(100/1/100) showed a peak that signifies the presence of the coupled species (i.e., (Li‐HT)^+^) at (1405) cm^−1^, while showing the TFSI^–^ peak that is significantly blue‐shifted from the pristine TFSI^−^ ((746) cm^−1^), as in the case of LT/HT(1/1). The overall phase behavior, with the data points of the two LT/FT/HT incorporated, is displayed in Figure S1, Supporting Information. The UV–vis results of LT/FT/HT, as compared with those of the binary mixtures, imply that the electron transfer from FT to HT is also present in the ternary mixture. Furthermore, the similarity of the peak intensity distribution between the two cases indicates the electron transfer of the ternary mixture is relatively unaffected by the presence of the ionic dopant. Similarly, it can be concluded that the ionic–electronic coupling between LT and HT is not significantly affected by the presence of FT in the ternary mixtures, based on the DSC and Raman results that did not show any pronounced spectral shifts or changes. To summarize the physical characterization of our ternary mixture system, it is believed that the following major doping effects are present; 1) the electronic doping effect where the charge transfer between FT and HT generates electronically active species NO˙ and facilitates the electronic conduction; 2) the ionic doping adds mobile ions (Li^+^) for the ionic conduction; 3) the ionic–electronic coupling between LT and HT ((Li‐HT)^+^) creates active species that contribute to the electronic conduction of the mixture. Of specific note is the orthogonality between the conduction pathways in our ternary system, where the electronic interaction (or charge transfer) is not significantly affected by the ionic interaction in the mixture. However, we note that there might exist possible interaction or coupling between the components or their derivatives when certain experimental conditions are met: For example, although we did not consider the coupling between the two dopants FT and LT, switching between the molar ratio in the mixture may lead to a coupled species that may affect the conduction behavior of the mixture significantly (Figure 5E).

**Figure 6:**
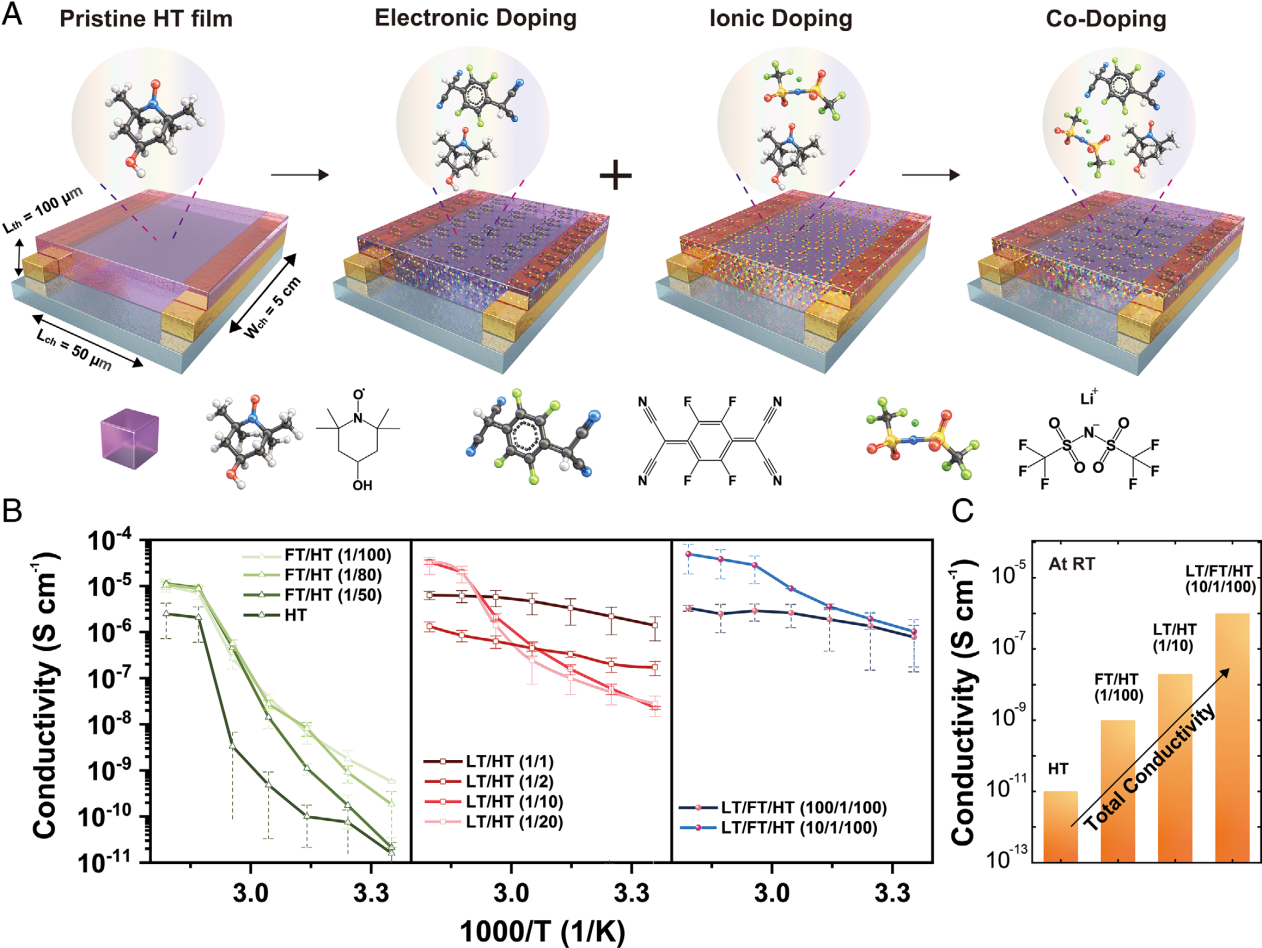
Electrical measurements of LT/FT/HT. A) Schematic illustration of the devices, showing the process of codoping in our system. B) Plots of the total conductivity (ionic + electronic) as a function of the temperature, for (left panel) LT/HT, (middle panel) FT/HT, and LT/FT/HT (right panel). The measured conductivity of the pristine HT is presented in the left panel (dark green). C) Total conductivity of HT, FT/HT (1/100), LT/HT (1/10), LT/FT/HT (10/1/100) at RT.

Based on the understanding of possible interaction and coupling between the components, we carried out electrical measurements to measure the effect of doping on the macroscopic electrical properties of the mixtures. We first measured the current–voltage (*I*–*V*) sweep to study how each dopant affects the conductivity of HT, and then compared the results with AC impedance for accuracy. For device fabrication, we deposited electrodes (Cr/Au = 10/50 nm) on a glass substrate using a thermal evaporator. We fabricated four different types of devices, each with different compositions (HT, LT/HT, FT/HT, and LT/FT/HT) (**Figure** **6**A). The devices were fabricated by drop‐casting of each mixture from the solution so that the resultant solid after drying connects the two electrodes. The bulk conductivity *σ* was calculated with the relation *σ* = (*LI*)(*WtV*)^−1^, where *W* is the width of the electrode, *L* is the channel length, *I* is the current, *V* is the voltage, and *t* is the thickness of the deposited solid. The devices were measured five times at each temperature for accuracy.

Figure 6 summarizes the overall trend of the measured (mixed) conductivity for all the devices fabricated. Previously, the pristine HT has shown a temperature‐dependent electrical behavior. Specifically, it displayed a very low conductivity at room temperature, but showed a sharp increase in the conductivity up to ≈2 × 10^−4^ S cm^−1^ at a channel length of 1.5 μm, above its *T*_m_. This was owing to the closer distance between the active radical sites of the pristine HT in the liquid phase, compared to the solid phase where it crystallizes to give a doubly intercalated structure.^[^ ^15^ ^]^ This time, we fixed the channel length of all the devices to be 50 μm, to test the mixed conduction through a long‐distance via possible long‐range order of the components in the mixture. In line with the previous report, pristine HT showed similar temperature dependence at the increased channel length (center panel, Figure 6B) but lower conductivity was observed because the different process is processed. The measured conductivity of the pristine HT was ≈10^−11^ S cm^−1^ at RT, while it increased to 5 × 10^−6^ S cm^−1^ at 80 °C (green line in middle column).

We first analyzed the effect of the electronic doping by FT on the conductivity of HT (left panel, Figure 6B). Electronic doping by FT showed similar temperature dependence to that of the pristine HT at all compositions tested (Figure 6B). The measurement was carried out based on the current‐voltage sweep of the two‐terminal structure and AC impedance (Figure S2, Supporting Information). The general conductivity trend was characterized by higher total conductivity compared to that of the pristine HT, for all the temperature range under observation. Noteworthy was the variation in the RT conductivity of FT/HT at different compositions, where the highest conductivity at 95 °C was found at FT/HT(1/50), with the measured conductivity of 0.3 × 10^−4^ S cm^−1^ through the AC impedance (Figure S2, Supporting Information). The increased overall conductivity of FT/HT is attributed to the generation of HT^+^ species that facilitate the charge transport, as discussed previously (Figure 5E). Here, the number of available HT^+^ species and its balance with the neutral HT is expected to be critical.^[^ ^14^, ^22^ ^]^ For example, adding FT above the ratio FT/HT(1/100) seems to interfere with the efficient charge transfer, as evidenced by the systematic increase in the RT conductivity of FT/HT at a higher molar ratio of FT (Figure 6B, Figure S1, Supporting Information).

Electronic doping by FT showed more complexity compared with that of LT. LT/HT devices fabricated showed higher RT conductivity at all compositions compared with that of the pristine HT, and showed the highest conductivity of up to ≈10^−5^ S cm^−1^ at LT/HT(1/1). (Figure 6B) In general, the electrical behavior of LT/HT was characterized by two distinct regimes with respect to the composition, with Regime 1 of low LT/HT and Regime 2 of high LT/HT ratio. In Regime 1, the conductivity trend showed similarity to that of the pristine HT, where it displayed the analogous temperature dependence albeit with much higher RT conductivity (left panel, Figure 6B). In contrast, Regime 2 showed a distinct conduction behavior, where the sharp transition upon temperature increase essentially disappeared. The transition from Regime 1 to Regime 2 as the ratio LT/HT increases is in accordance with the characteristic phase transition of LT/HT observed in the DSC and Raman results (Figure 3 and 4) and presents solid evidence on the connection between the phase of LT/HT and its electrical behavior. Noteworthy is the RT conductivity of the LT/HT in Regime 2 being rather higher than that of Regime **1**, indicating possible modulation of the conductivity by switching the doping ratio on‐demand. Importantly, the lowest activation energy was observed for LT/HT(1/1), which also correlates with the thermodynamic characterization made previously (Figure 3 and 4).

Qualitatively, the measured conductivity of the ternary mixture is summarized as a linear combination of the results from the two separate doping systems, LT/HT and FT/HT (right panel, Figure 6B). For example, LT/FT/HT(10/1/100) displayed the conductivity modulation similar to the case of LT/HT(1/10) (Regime 1), while showing the higher values of conductivity at all the temperature range, which is the characteristics of the FT/HT system. As a result, LT/FT/HT showed the highest mixed conductivity among all the samples tested, where the conductivity of 0.8 × 10^−4^ S cm^−1^ was obtained at 95 °C in DC measurement. Noteworthy is that the observed conductivity for LT/FT/HT(10/1/100) was generally higher than that of LT/FT/HT(100/1/100). It is attributed to the charge transport dominated by the ionic conduction due to a large amount of LT present in the system, which significantly hinders the conduction through the electronic pathway. Remarkably, the key advantage of the ternary system was found in the lower temperature range, especially at RT (Figure 6C). Starting from the pristine HT, a conductivity increase of approximately five orders of magnitude was found in LT/FT/HT(10/1/100). Specifically, the observed increase was a sum of the doping effects from FT and LT, which increased the mixed conductivity by approximately two‐ and three orders of magnitude, respectively.

**Figure 7:**
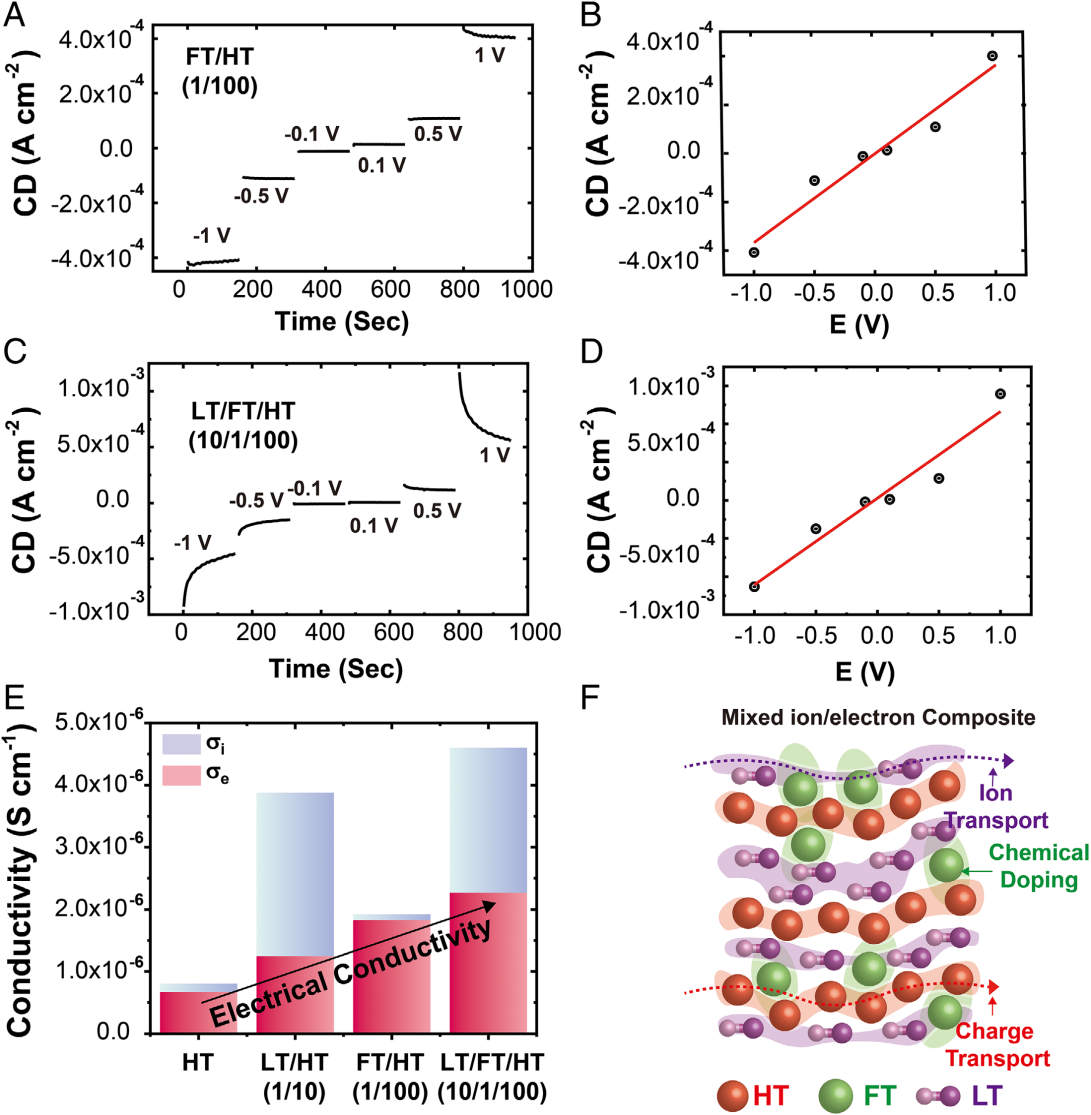
Decoupled conductivity measurements of the mixed conductors. Current variation curve as a function of time at various applied DC potentials for A) FT/HT(1/100), B) with corresponding Ohm's law plot, and C) LT/FT/HT(10/1/100), D) with its Ohm's law plot. E) Decoupled conductivity analysis displaying the contribution from each transport pathway. F) Schematic illustration of the mixed conduction in LT/FT/HT.

For more quantitative assessment on the observed coupling in our codoping system, we conducted a DC polarization experiment on the mixtures above *T*_m_. It allows the measurement of the contribution from each conduction pathway to the total mixed conductivity and is described in **Figure** **7**. During the DC polarization, the pristine HT and the mixtures exhibited steady‐state current after the initial convergence, owing to the ionic transport that decays over time (Figure 7A,C). The resultant steady‐state current density was plotted as a function of applied voltage in Figure 7B,D. Figure 7E shows the summary of the ionic and electronic contribution to the total mixed conductivity above *T*_m_, derived from the DC polarization plot and AC impedance results. In the plot, the pristine HT mainly exhibits electronic conduction owing to the radical conduction. In contrast, the ionic doping (i.e., LT/HT) resulted in a significant increase in the ionic conduction of the mixture, resulting in a total conductivity of 0.4 × 10^−5^ S cm^−1^ at 70 °C. The ionic doping also involved an appreciable increase in the electronic conduction, owing to the ionic–electronic coupling between LT and HT that forms (Li‐HT)^+^ (vide supra).^[^ ^14^ ^]^ In FT/HT, the electronic doping by FT mainly resulted in an increase in the electronic conduction by the charge transfer between FT and HT. Notably, the highest mixed conductivity found in LT/FT/HT(10/1/100) (0.45 × 10^−5^ S cm^−1^ by DC polarization, 1.2 × 10^−4^ S cm^−1^ by AC impedance) was achieved by the significant contributions from both pathways, where all the aforementioned doping effects contributed quantitatively.

Overall, the analysis of the contribution from each transport pathway in our ternary mixture system provides a deeper insight into its conduction behavior. Specifically, the fact that the mixed conductivity is expressed as a sum of all the doping effects at both high and low temperatures indicates the orthogonality between the dopants and their derivatives in our system. For example, the formation of HT^+^ by the addition of FT does not significantly affect the ionic–electronic coupling between HT and LT above *T*_m_. This is also supported by the results from the physical characterization methods as discussed above. We believe it is related to the general characteristics of the doping process, where only a small amount of dopant addition enhances the transport efficiency greatly. Because it only consists of a small portion in the mixture, it does not seem to affect the other equilibrium processes in the mixture. Although we emphasized the decoupled analysis of the ternary mixture at high temperatures, we note that a similar trend of decoupling between each dopant was also found at RT (Figure 6C). It enabled the modulation of the mixed conductivity up to five orders of magnitude, by the sequential doping of FT and LT, where the effect of doping from each could also be controlled strictly on demand.

**Figure 8:**
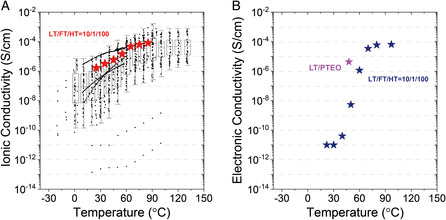
State‐of‐the‐art comparison of the reported conductivity. A) The ionic conductivity and B) electronic conductivity reported for the LT/PTEO system, as a function of temperature.^[^ ^23^ ^]^ The conductivities of LT/FT/HT(10/1/100) in this work are marked as red and blue stars, respectively.

We compared the performance of our champion device with the current state‐of‐the‐art: ionic and electronic conductivities of LT‐doped PTEO (LT/PTEO) as a function of temperature. The average ionic conductivity of LT/FT/HT(10/1/100) was comparable to that of LT/PTEO, in a temperature range of 25–100 °C (**Figure** **8**A). Further, the electronic conductivity of LT/FT/HT outperformed that of LT/PTEO at a high‐temperature regime (Figure 8B), where the device showed stable operation with negligible hysteresis (not shown here). We emphasize the stable operation at relatively high conductivities observed in our system has rarely been reported among the conducting organics, and we believe our design principle of mixed conduction system represents the true potential of the open‐shell chemistry necessary for the next‐generation organic conductors.

## 3 Conclusion

As an emerging class of conducting organics, small molecular radicals have shown substantial promises. In this work, we expanded the conventional idea of molecular doping into a more generalized concept of doping organic radicals. We explicitly discussed the factors affecting the coupling between the components, and their contribution to the mixed conductivity. We thereby postulated a charge transport mechanism associated with the codoping system, the knowledge from which can potentially be applicable to future electronic/ionic circuits. We believe our findings represent a versatile and robust chemical design principle by which to engineer the open‐shell chemistry of organic electronics, which opens the door for the future generation of mixed ionic and electronic conductors.

## Conflict of Interest

The authors declare no conflict of interest.

## Data Availability Statement

Research data are not shared.

## Supporting information

Supplementary Material
