# Supplementary material for: Sequential Codoping Making Nonconjugated Organic Radicals Conduct Ionically Electronically
Source: Small Sci. 2021 Oct 4;2(1):2100081. doi: 10.1002/smsc.202100081 (PMC11936003; doi:10.1002/smsc.202100081)
Supplement: Supplementary file 1 — Supplementary Material [file SMSC-2-2100081-s001.pdf]

Supporting Information

Sequential Co-Doping Making Non-Conjugated Organic Radicals Conduct Ionically-Electronically

Yerin Jo,[1,†,‡] Ilhwan Yu,[2,†,‡] Jaehyoung Ko,[3,†,‡] Ji Eon Kwon,[†] Yongho Joo[†,*]

[†] Institute of Advanced Composite Materials, Korea Institute of Science and Technology (KIST), 92 Chudong-ro, Bongdong-eup, Wanju-gun, Jeonbuk 55324, Republic of Korea
[1] Department of Nanoconvergence Engineering, Jeonbuk National University, 567 Baekje-daero, Deokjin-gu, Jeonju-si, Jeonbuk, 54896, Republic of Korea
[2] Department of Chemistry, Hanyang University, 222 Wangsimni-ro, Seoul 04763, Republic of Korea
[3] Department of Chemical and Biomolecular Engineering and KAIST Institute for Nano Century, Korea
Advanced Institute of Science and Technology (KAIST), Daejeon 34141, Korea
[*] To whom correspondence should be addressed, yjoo0727@kist.re.kr
[‡] These authors contributed equally to this work.

Table of contents

Experimental Section

Figure S1

Figure S2

Figure S3

Experimental section

Materials and methods

All chemicals were purchased from commercial sources and used without further purification otherwise noted. These include 4-hydroxy-2,2,6,6-tetramethylpiperidin-1-oxyl (> 98%, Tokyo Chemical Industry), bis(trifluoromethylsulfonyl)amine lithium (> 99.0%, Sigma-Aldrich), and 2,3,5,6-Tetrafluoro-7,7,8,8-tetracyanoquinodimethane (> 99%, Ossila). The abbreviated notation for each compound described below follows the same convention as described in the main text.

To prepare the doped HT mixtures (LT/HT, FT/HT, and LT/FT/HT), stock solutions of each component were prepared first in acetone (HT, LT, and FT, the stock concentration of 10, 10, and 1 mg mL^{-1}, respectively). These stocks were then mixed in varying amounts to achieve the desired molar ratio between the components. The molar ratio of LT/HT tested in this study includes 1/1, 1/2, 1/3, 1/4, 1/5, 1/6, 1/7, 1/8, 1/9, 1/10, 1/11, 1/12, 1/15, and 1/20. For FT/HT, the mixtures of molar ratios 1/50, 1/80, and 1/100 were tested. For LT/FT, the mixtures of molar ratios 1/2, 1/5, 1/8, 1/10, 1/20, 1/50, 1/80, and 1/100 were tested. Lastly, for LT/FT/HT, the mixtures of the ratio 10/1/100 and 100/1/100 were prepared. After the mixing, each sample was homogenized with bath sonication (JAC-3010, Kodo technical Research, 490 W) for 10 min. The resultant solutions were characterized by a set of spectroscopies. For the measurements in a solid state, the samples were prepared by evaporating the solutions in a vacuum oven at room temperature overnight.

Before the device fabrication, all substrates were cleaned by bath sonication in acetone, 2-propanol, and chloroform for 10 minutes each. The substrates of all devices used a slide glass (Paul Marienfeld GmbH & Co. KG**,** Germany) with a thickness of 1 mm and a channel width

of 5 cm. The devices were fabricated by first depositing Cr (10 nm) and Au (50 nm) on slide glass via a thermal evaporator, with a defined channel length (50 μm) using appropriate masks. It was followed by a drop-casting of a sample solution in acetone (concentration 5 mg mL^{-1}), which was then dried in the air overnight for the channel formation. For the sample solutions to be cast, the pristine HT and the doped mixture solutions prepared above were used. Thermal annealing on a hot plate at 85℃ was carried out for all the devices prepared, for the better definition of the channel. The thickness of the pristine HT and the solid mixtures on the devices was measured on a surface profiler (ET200, Kosaka, Japan). All mixtures were measured to have an approximate average thickness of 100 μm.

Characterization

Current-voltage (I-V) profiles were acquired for the devices with a sweeping voltage in the range of $-1\ V \le V \le +1\ V$ using an electrometer (Keithley 4200, Tektronix). It was also utilized in measuring the electrical conductivity in a temperature range of 298 K < T < 358 K. The electrical conductivity was calculated based on the following equation:

$$\sigma = \frac{1}{R} \times \frac{l}{A}$$

where R is the electrical resistance, l is the channel length (50 μm), A is the channel area. DC polarization experiments were obtained by measuring the current with time using an electrometer (Keithley 4200, Tektronix) at -1, -0.5, -0.1, 0.1, 0.5, and 1 V voltages for 150 s each. Electrochemical impedance spectroscopy (EIS) of the devices was carried out on a potentiostat (Interface 1010E, Gamry). 2 MHz to 0.1 Hz of frequency range was applied, with an oscillating peak potential of 100 mV. The samples were equilibrated at 75 °C for 5 minutes before each measurement. The ionic conductivity was calculated by the relation $\sigma_{ion} = d\ /\ (R_{ion}A)$, where R_{ion} is the bulk ionic resistance of the electrolyte, σ_{ion} the ionic

conductivity, *d* the distance between the electrodes, and *A* the area between the electrolyte and the electrodes. UV-Vis spectra of the pristine HT and the mixtures were collected using a multipurpose UV/Vis/NIR spectrophotometer (V-670, Jasco). All mixtures containing FT were measured in acetone solution, and LT/HT mixtures without FT were measured in an aqueous solution. A quartz cell of 10 mm pathlength was used, and a dual-beam setup was applied. Electron paramagnetic resonance (EPR) spectra of the solutions were collected through an EPR spectrometer (JES-FA100, JEOL). For the measurement, 1 mL of the mixture solutions was transferred to a glass tube, and the measurement was carried out at RT. Differential scanning calorimetry (DSC) was carried out on a differential calorimeter (Q20, TA Instruments) at a temperature range from -90 °C to 80 °C, with a step of 2 °C/min in the nitrogen atmosphere. For each measurement, the solid mixture (approximately 5 - 10 mg) was transferred to an aluminum pan. The T_g and T_m of all mixtures were determined using a single heating cycle. Raman spectra of the mixture solutions were collected on a multipurpose Raman spectrometer (Labram, Horiba). A green laser of 514 nm wavelength (50 mW) was used for the excitation. **DFT calculation**

All density functional theory (DFT) calculations were performed in the gas phase using the Gaussian16 quantum-chemical software package. The unconstrained geometry optimizations for the molecules were performed using the (U)B3LYP functional and 6-31g+(d,p) basis set. Vibrational frequency calculations were performed for the obtained structures at the same level to confirm the stable minima.

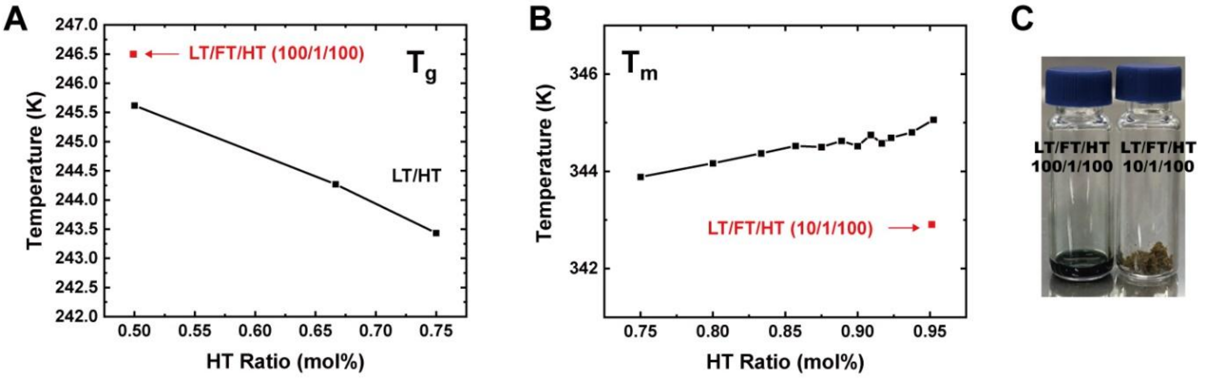

Figure S1. Experimental phase diagram of LT/HT and LT/FT/HT displaying (A) T_g and (B) T_m from DSC measurements. (C) Photograph of LT/FT/HT showing the liquid nature of LT/FT/HT(100/1/100).

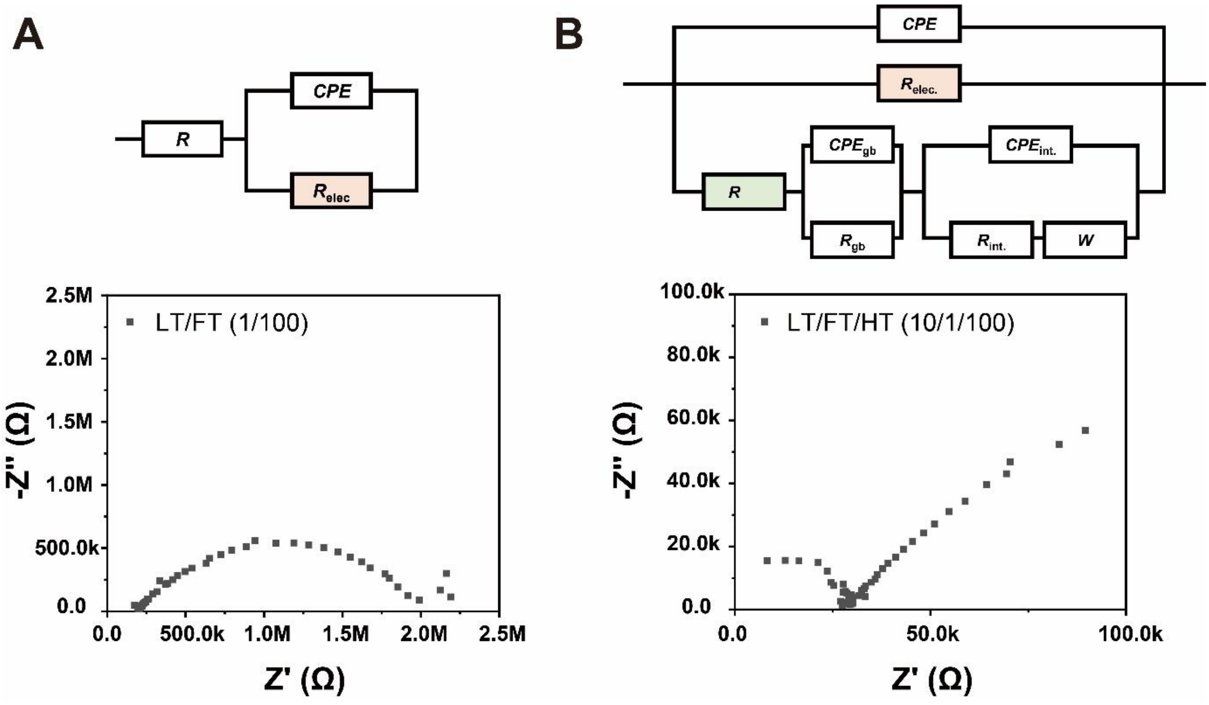

Figure S2. AC impedance of (A) LT/FT(1/100) and (B) LT/FT/HT(10/1/100), showing the electronic conduction and ionic-electronic conduction, respectively.

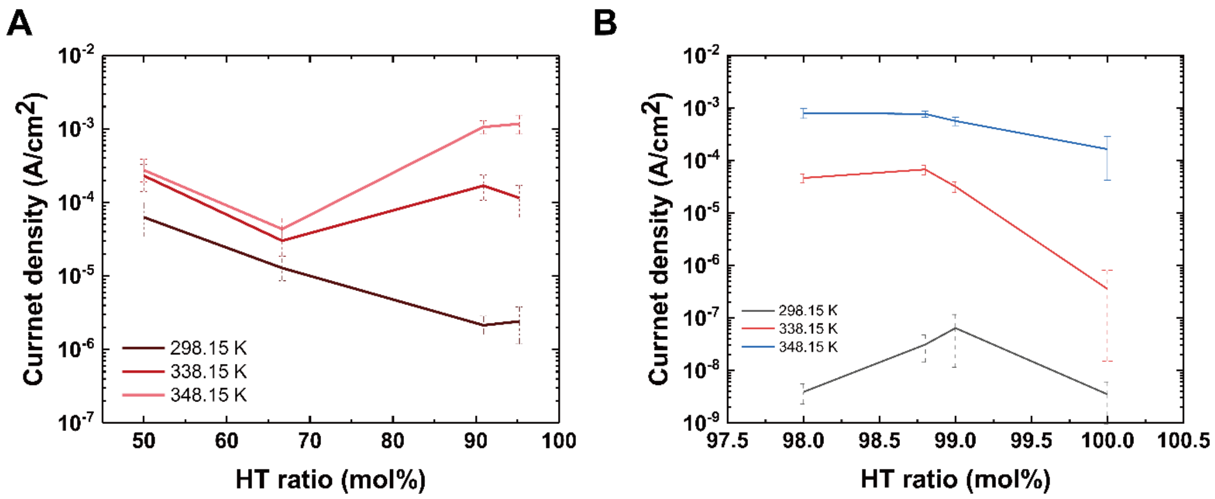

Figure S3. Current density plot of (A) FT/HT, and (B) LT/HT as a function of the HT ratio.
